# Microscopic anatomy of the lungs of domestic animals, mice, and rats

**DOI:** 10.1177/10406387251413159

**Published:** 2026-01-21

**Authors:** Kathleen R. Mulka, Rachael C. Gruenwald, Tzushan Sharon Yang, Jeff L. Caswell

**Affiliations:** University of Pennsylvaniaa School of Veterinary Medicine, Philadelphia, PA, USA; Oregon Veterinary Diagnostic Laboratory, Oregon State University, Corvallis, OR, USA; Department of Pathology Microbiology and Immunology, Vanderbilt University Medical Center, Nashville, TN, USA; Department of Pathobiology, University of Guelph, Guelph, ON, Canada

**Keywords:** comparative pathology, distal airways, histology, lungs, pulmonary circulation

## Abstract

The lung is composed of conducting airways, a gas-exchange region, and a dual circulatory system. Any of these components may be altered in respiratory disease, and complicated cases can be a diagnostic challenge. For veterinary pathologists, a solid foundation in normal anatomy is essential for recognition of patterns of disease. Additionally, the structure of the lungs informs the function; therefore, knowledge of how normal structures are disrupted provides insight into the pathogenesis of lung diseases. We detail the organizational structure, microanatomy, and cell types of the lungs of several species of veterinary importance: cattle, horses, pigs, sheep, goats, dogs, cats, mice, and rats. Animals with a thick pleura and interlobular septa have associated separation of secondary lobules, whereas those with a thin pleura lack interlobular septa and have indiscernible secondary lobules. The transition between terminal bronchioles and gas-exchange regions, presence of respiratory bronchioles, and cellular composition of the bronchioles are highly variable among species. Other species variations include bronchial structure and glands, collateral ventilation, and patterns of blood supply to the conducting airways, gas-exchange regions, and pleura. Examples of histopathologic correlates offer relevance of pulmonary microanatomy to the veterinary pathologist.

Lungs serve the essential function of providing a conduit for gas exchange, which allows for oxygenation of tissues. Many specialized cell types and structures within the lungs contribute to this main function and provide a means of defense and homeostatic regulation. There are species differences in the cellular and structural composition of the lungs. For veterinary pathologists, knowledge of how normal structures are disrupted offers insight into the pathogenesis of lung diseases. Understanding the normal microanatomy of the lower respiratory tract is the basis for better classification of pulmonary disease in veterinary species, and for the development of animal models of human disease.

Detailed studies of pulmonary anatomy have traditionally been conducted using histology, microdissection, latex injection of vessels, and electron microscopy.^35,45,52,59^ Knowledge of cell type composition has been gained through analysis of cellular morphology and immunohistochemistry. New and emerging techniques, including single-cell RNA sequencing and spatial transcriptomics, are revolutionizing the understanding of cell types in normal and disease conditions based on their broad patterns of gene expression, and many such studies are publicly available in the Lung Cell Atlas^61^ and LungMAP.^12^ This approach has strong potential for differentiating functional subtypes of known cells, understanding how cells differentiate, and identifying new cell types. This type of information may be applicable to understanding disease, identifying biomarkers, and selecting targets for treatment.^21^ Most of these studies have been conducted using human and mouse tissues,^9,68^ but data in other veterinary species are emerging.^11,27,54,57,58,65^ Species differences in cell type are relevant for understanding species-specific disease and offer context for the application of animal models of human disease.

The purpose of our review is to provide a reference for the comparative microanatomy of the lungs of several domestic and laboratory animal species, with a focus on distal airways, gas-exchange region, and circulation of the lungs. Additionally, examples of histologic correlates are described to emphasize relevance to the veterinary pathologist.

## Organization and function of the lung

The main function of the lung is gas exchange, allowing oxygen to enter and carbon dioxide to be removed from the circulating blood,^81^ which helps deliver nutrients to tissues and maintain acid–base balance. In carrying out this task, the respiratory system is continuously exposed to the external environment. Therefore, additional lung functions must include surveillance for noxious or infectious stimuli and a means of defense against them. The lung is divided into conducting and gas-exchange components, which are surrounded and variably divided by interlobular connective tissue and pleura. Conducting airways facilitate entry and exit of air to the gas-exchange region. They also warm and humidify the incoming air, remove many particulates by impaction, and facilitate immune responses to inhaled antigens.^85^ Within the lung, bronchi and bronchioles comprise the conducting components; the gas-exchange region is composed of alveoli and alveolar ducts, and respiratory bronchioles (in some species). There are about 15 generations of conducting airways and about 8 generations of gas exchange spaces, with considerable species and individual differences.^76^

Organizational units of the lungs described in the literature include lobes, lobules, bronchopulmonary segments, and pulmonary acini. Understanding the different organizational units can be beneficial in identifying and communicating patterns of disease. A *lung lobe* is the region of lung ventilated by a single secondary bronchus (or for the right cranial lobe of pigs and ruminants, by the tracheal bronchus that arises directly from the trachea proximal to its bifurcation). A *bronchopulmonary segment* is the region of lung supplied by the tertiary or segmental bronchi. The definitions of smaller components of the lung have been established by their use in humans, but the anatomy of domestic animals has key differences that cause discrepancy in how these terms are used in other species. The *pulmonary acinus* is a functional and structural unit of the lung that is defined as the region ventilated by a single conducting airway. Specifically, in species with well-developed respiratory bronchioles, the pulmonary acinus is defined as all tissue including and distal to the transitional bronchiole. The *transitional bronchiole* refers to the bronchiole in which the first or most proximal alveolar outpouching occurs.^78^ In species with poorly developed respiratory bronchioles, this can be approximated by the terminal bronchiole itself. Because there is species variation in the extent of respiratory bronchioles (see below), the size of the pulmonary acinus varies among species.^45^

Terminology around pulmonary lobules is more variable. The *primary lobule* is defined as the area supplied by one respiratory bronchiole,^84^ or the alveolar duct and all distal structures^8,15^; however, this structure is not identifiable by diagnostic imaging and is not routinely used.^15^ The term “lobule” generally refers to “secondary lobule.” Secondary lobules are not defined by the airways that supply them but rather by the connective tissue surrounding them. As defined in Nomina Histologica Veterinaria,^84^ the *secondary pulmonary lobule* includes the terminal bronchiole and its associated pulmonary acini.

A dual circulatory system supplies the lungs. The *pulmonary circulation* primarily serves to perfuse the lung for gas exchange and is a high-capacity, low-resistance system receiving the entire cardiac output from the right ventricle. The *bronchial system* is part of the systemic circulation and gives nourishment to the bronchioles and bronchi (and other lung structures in some species), receiving a small percentage of oxygenated blood from the left ventricle within a low-capacity, high-resistance system.^8,23^ These 2 blood supplies are discussed in further detail below.

Within the lung, the greatest species variation occurs in the number of lobes, lobules, the presence and thickness of pleura and interlobular septa, the composition of the terminal conducting airways, the structures supplied by the bronchial versus pulmonary circulation, and the constitutive presence of pulmonary intravascular macrophages.^35,45^

## Pleura and interlobular septa

**Figure 1.**
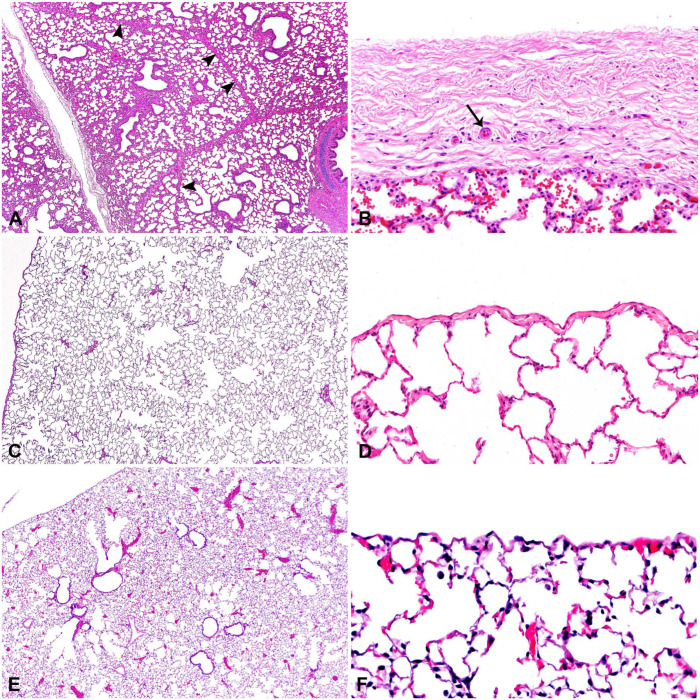
Difference in pulmonary septation and pleura of representative species. **A.** Pig interlobular septa (arrowheads) are thick and surround pulmonary lobules. **B.** Horse pleura is composed of thick connective tissue, occasionally interspersed with small arterioles (arrow). **C.** Dog pulmonary parenchyma lacks septation and lobules. **D.** Dog pleura is thin compared to large animals. **E.** Mouse pulmonary parenchyma lacks septation. **F.** Mouse pleura is thin, and connective tissue layer is not noticeable on high magnification.

The *pulmonary (visceral) pleura*, a serous membrane that covers the outer surface of the lungs, is composed of mesothelial cells, connective tissue, and elastic layers.^74^ The thickness of the pleura is variable among species and between different regions of the lung within species. For example, in cattle and horses, the pleura is thicker in caudodorsal compared with cranioventral regions of lung and should not be confused with a fibrotic pleural disease. Horses, sheep, cattle, and pigs have a thick pleura, whereas dogs and cats have a thin pleura.^35^ In mice and rats, the visceral pleura includes the same components as in other species, but is extremely thin and nearly inappreciable by light microscopy (**Table 1; Fig. 1, Suppl. Figs. 1–8**).^38,44^

**Table 1.** Comparison of lung structure among several species of veterinary importance.

Structure	Type I			Type II		Variant type II		Type III
	Cattle	Sheep	Pig	Dog	Cat	Mouse	Rat	Horse
Pleura	Thick	Thick	Thick	Thin	Thin	Extremely thin	Extremely thin	Thick
Interlobular septa	Complete	Complete	Complete	−	−	−	−	Incomplete
Respiratory bronchioles	−	−	−	+	+	Poorly developed	Poorly developed	−

+ = present; **−** = absent.

*Interlobular septa* also vary in thickness and completeness between species. Prominent and thick connective tissue septa surround and separate pulmonary lobules in cattle, sheep, and pigs. In horses, the septa are thick but incomplete. Dogs, cats, mice, and rats do not have interlobular septa (Table 1; Fig. 1, Suppl. Figs. 1–8).^35,45,72^

## Histopathology correlate: interlobular septa

**Figure 2.**
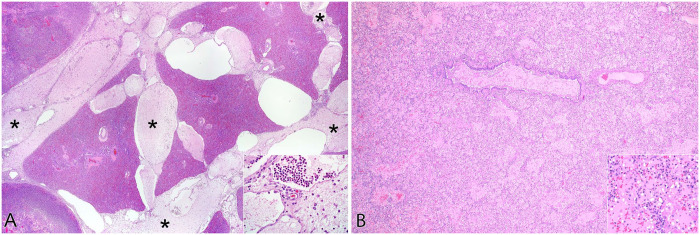
Interlobular septa vary in thickness and completeness between species. **A.** Calf with *Mannheimia haemolytica* pleuropneumonia; interlobular septa (*) are expanded by edema, neutrophils, and distended lymphatics. Inset: higher magnification of interlobular septa. **B.** Dogs do not have interlobular septa. In an inflammatory process resulting in interstitial and alveolar edema and fibrin accumulation, interlobular lesions are not apparent. Inset: higher magnification of alveolar septa and spaces.

In cattle, pigs, and horses, the interlobular septa are often expanded by edema in cases of left-sided heart failure, by fibrin and edema in cases with acute fibrinous pleuritis, and by emphysema and edema in cases of interstitial lung disease with respiratory distress. In contrast, these interlobular septal lesions are not evident in dogs, cats, mice, and rats with similar conditions (**Fig. 2**).^6,35^

## Conducting airways—bronchi

The airways form a progressively branching system from the trachea to the bronchioles.^56,78,81^ The distal portion of the trachea diverges into 2 *mainstem* or *primary bronchi*, which further divide into *lobar* or *secondary bronchi* and then *segmental* or *tertiary bronchi*. Branching beyond this point generates *subsegmental bronchi*.

In the literature, *airway branching patterns* are defined by the number of branches arising from a single parent airway, the size of the daughter branches, and the symmetry with which branching occurs. Slightly different definitions are encountered when referring to these parameters, which makes comparative analysis challenging. In some references, dichotomous branching refers to each airway tube giving rise to 2 daughter branches.^46,78^ By this definition, all mammalian lungs are dichotomous. In other references, a dichotomous branching system refers to the parent airway dividing into 2 smaller daughter segments of approximately equal diameter, whereas monopodial branching refers to the parent airway dividing into daughter airways of unequal diameter.^14,38,45^ The airways of most species branch in an asymmetric fashion of various degrees, with humans being the most symmetric.^46^ The architecture and branching pattern of bronchi are major determinants of the distribution of inhaled particles, with implications for health and disease.^46^ Further information on airway branching and morphometry can be found in published comprehensive reviews.^46,78^

**Figure 3.**
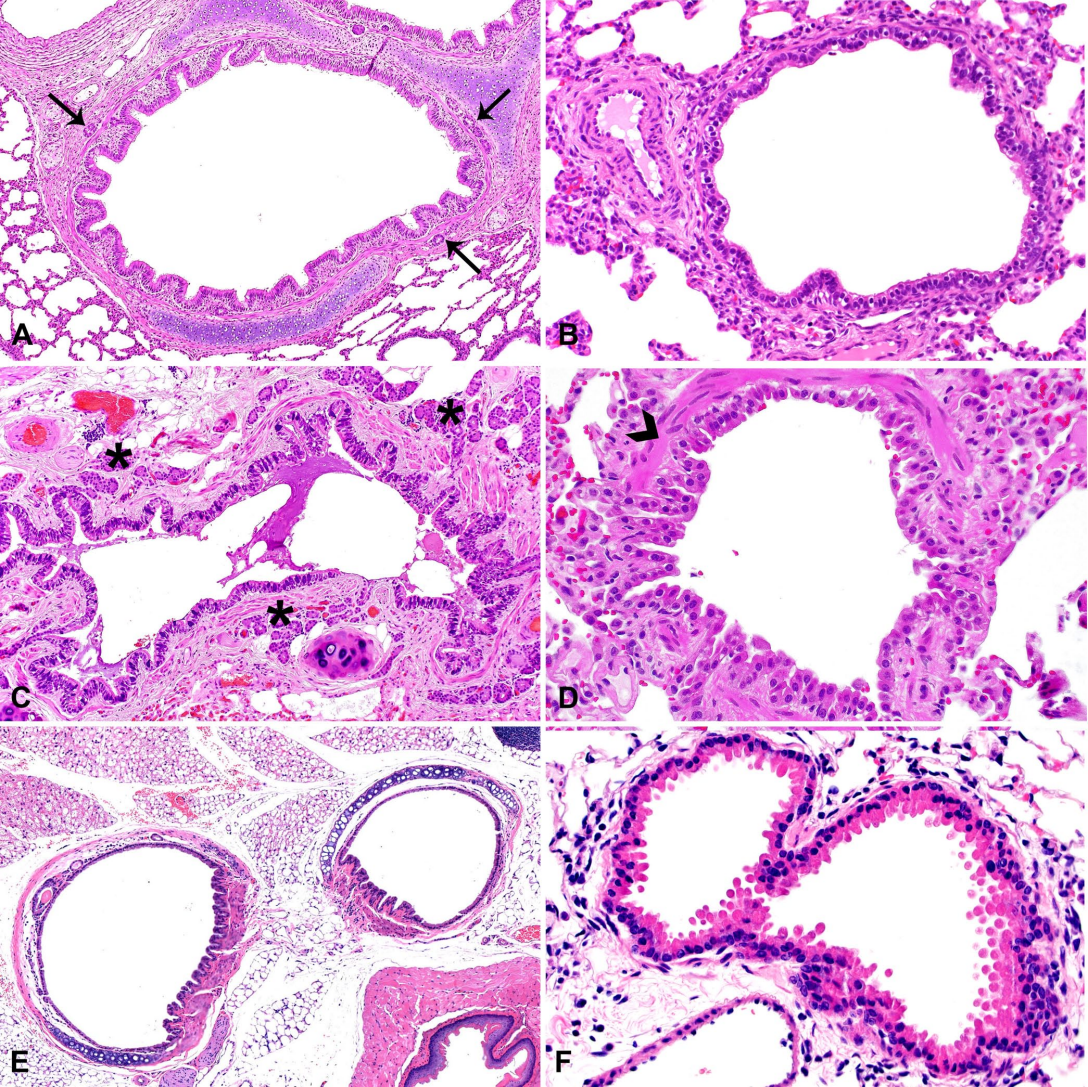
Conducting airways of representative species. **A.** Pig bronchus supported by hyaline cartilage and surrounded by mucosal glands (arrows). **B.** Pig bronchiole lined by pseudostratified columnar ciliated epithelium. **C.** Cat bronchus supported by hyaline cartilage with clusters of mucosal glands (asterisks) throughout the adjacent lamina propria. **D.** Cat bronchiole lined by pseudostratified columnar ciliated epithelium and supported by smooth muscle (arrowhead). **E.** Mouse bronchi; only extrapulmonary bronchi are present, which lack mucosal glands. **F.** Mouse bronchioles lined by a mixture of club cells and ciliated epithelium with no mucosal glands present.

*Bronchi* are composed of respiratory mucosa, a fibromusculocartilaginous tunic, and adventitia (**Fig. 3**). The bronchial mucosa includes the lamina epithelialis, lamina propria, bronchial glands, and bronchial lymphatic nodules (also referred to as bronchus-associated lymphoid tissue or BALT). The lamina propria is indistinct and features reticular, elastic, and collagenous fibers, along with vascular capillary channels. The fibromusculocartilaginous tunic consists of the smooth muscle layer and bronchial cartilage.^84^ Unlike most domestic mammals, mice and rats technically lack intrapulmonary bronchi, because all conducting airways within the pulmonary parenchyma lack cartilage (Suppl. Figs. 7, 8). We agree with this characterization and recommend the use of the term bronchioles to describe intrapulmonary airways in mice and rats. However, numerous studies describe intrapulmonary airways in these species as bronchi rather than bronchioles. It is important to be aware of this discrepancy when researching these species.^43^

Proximally, in addition to conducting air flow, bronchial functions include purification and humidification of inspired air and detection of inhaled noxious substances. Epithelial cells defend against potentially harmful agents by creating an intact surface barrier, secreting antimicrobial substances, and effectively cleaning the airways through mucociliary clearance.^8^ Two layers make up the airway surface liquid—a *mucus layer* and *periciliary liquid layer*. The mucus layer has higher viscosity, with the ability to bind and trap inhaled particles, and it contains antimicrobial substances. The periciliary liquid layer has lower viscosity, which allows cilia to beat through it and clear the overlying mucus.^24^ Numerous epithelial cell types have been characterized in the surface epithelium and bronchial glands, each expressing specific genes or proteins (**Table 2**). Ultrastructural features and functions of all of these cell types have been reviewed extensively.^9,56^ Each cell is specialized to perform essential functions within the airways.

**Table 2.** Markers for pulmonary epithelial cells by RNA sequencing and immunohistochemistry.

Cell type	Cell marker	Ref.
Ciliated cells	CCDC113, CCDC17, FOXJ1, DNAI1, CYP4B1	^28,39,54^
Basal cells	p63, p40, cytokeratin 5/6	^60,65^
Mucous or goblet cells	MUC5AC, MUC5B, Tff2	^37^
Pulmonary neuroendocrine cells	Chromogranin A	^2,40^
Brush or tuft cells	Dclk1, Gnat3, Trpm5	^2,39^
Ionocytes	FOXI1, CTFR, Barttin (BSND), Ascl3	^65^
Club cells	SCGB1A1, surfactant protein A	^22^
Alveolar type I cell	Cytokeratin 7, cytokeratin 19, AGER	^22,54^
Alveolar type II cell	TTF1, surfactant apoprotein Aa, napsin A, LAMP3, cytokeratin 8, cytokeratin 18	^22,28,54^

The proximal airways are lined by pseudostratified epithelium. In general, the epithelium is tall columnar proximally and becomes more cuboidal distally. In the bronchi, frequently identified cell types within the lamina epithelialis include *ciliated cells, basal cells*, and *goblet/mucous cells*. Ciliated cells are tall columnar cells with apical cilia and microvilli. They move mucus through the rapid beating of apical cilia and facilitate clearance of mucus and entrapped particles, debris, and microorganisms.^9^ Basal cells are cuboidal with scant amounts of cytoplasm and round nuclei. Basal cells help anchor the epithelium by strongly adhering to the basement membrane and to adjacent columnar cells, support homeostasis, and act as stem cells. Basal cells can differentiate into goblet, club, ciliated, brush or tuft, pulmonary neuroendocrine cells, and pulmonary ionocytes.^9,60^ Mucous (goblet) cells are tall columnar cells that variably contain a large secretory vacuole, distending the apical cytoplasm, depending on the status in the secretory cycle.^8,9,19^ Mucous cells are the main mucus-producing cells of the surface epithelium. Mucociliary clearance is a main defense mechanism of the lung, and the ideal viscosity and amount of mucus are essential.

Ciliated, basal, and mucous cells have been detected and described in histologic and ultrastructural investigations in cattle, horses, pigs, sheep, dogs, cats, mice, and rats.^3,5,18,71^ The literature often describes epithelial cells in terms of tracheobronchial epithelium, without specific characterization of bronchi. Studies on the percentage composition of each cell type within the bronchi have been conducted in sheep, but not in other species covered in our review.^30^ Single-cell RNA sequencing in canine^58^ and porcine^65^ lungs has also characterized these cell types and provided additional gene expression profiles. The canine study was performed on dissociated whole lung tissue; hence, more specific localization was not possible (i.e., epithelial cells of the proximal versus distal airways).^58^ The porcine study used microdissection and confirmatory immunohistochemistry; basal, secretory, and ciliated cells with the same gene expression markers and morphology had different transcriptional profiles, depending on their location in either small or large airways.^65^

Cell types infrequently identified in the surface epithelium include *pulmonary neuroendocrine cells (* ***PNEC****)*, *brush* or *tuft cells*, and *pulmonary ionocytes*. PNECs are bottle-shaped with a small, round nucleus and granulated cytoplasm. These cells are located throughout the airways, most frequently at bifurcations of the bronchi and bronchioles. They can be single cells or grouped, and the latter are referred to as *neuroendocrine bodies*. PNECs are surveillance cells that secrete neuropeptides to signal the immune system.^8,9,40,56^ Brush or tuft cells have apical clustered densely packed microvilli and are located in many organs in addition to the lung.^9,56^ Within the respiratory system, these cells have been described in the trachea and bronchi of several species, and in the alveoli of rats. Brush or tuft cells have chemosensory, neuronal, and immunologic functions. Pulmonary ionocytes have been identified through single-cell RNA sequencing and described in mice and humans^9,39^; they function in fluid regulation and mucus clearance.

In histologic and ultrastructural studies, PNECs have been identified in pigs, sheep, dogs, cats, mice, and rats.^18,26,75^ Brush or tuft cells have been identified in cattle, horses, pigs, sheep, cats, mice, and rats.^9,18,41,56^

Mucosal glands (bronchial glands) are present in greatest numbers at airway bifurcations.^83^ Cell types in the mucosal glands include *basal cells*, *mucous cells*, *serous cells*, *seromucous cells*, *duct cells*, and *myoepithelial cells*. Mucous, serous, and seromucous cells are differentiated based on ultrastructural characteristics and the content of secretory granules. Four different types of mucous cells have been described in sheep.^30^ In humans and pigs, these cells have also been defined by patterns of gene expression.^61,65^ In mice, mucous cell types have been identified based on single-cell transcriptomic studies, but many studies have one category for all secretory cells.^12,33^

Various species have different combinations of cells that contribute to the production of airway surface liquid. Cells involved in mucus production include mucous cells on the epithelial surface and mucous and serous cells within mucosal glands. Surface serous cells have been reported in specific pathogen–free (SPF) rats and some cats, but are mainly considered to be a cell of the mucosal glands.^56^ Some species have prominent and well-developed mucous glands and fewer mucous cells on the epithelial surface, whereas other species have few or no mucous glands and a greater number of surface secretory cells. Cattle, pigs, sheep, and dogs have abundant mucosal glands in the proximal trachea and fewer glands in the bronchi. Cats have abundant mucosal glands in the proximal trachea and bronchi, and glands also can be detected in the bronchioles. Horses have mucosal glands in the trachea and bronchi, but in lower abundance compared with other species.^18^ In mice and rats, a few mucosal glands are present in the proximal trachea, but are completely absent in the lower conducting airways.^82^ Mucous cells in mice are present in the largest numbers in the trachea and proximal airways. In one study, none were found in intrapulmonary airways.^43^ In general, mucous cells in mice and rats are infrequent compared with larger mammalian species, but may increase in number in the face of inflammation or infection.^38^

## Histopathology correlate: mucous cell hyperplasia and metaplasia

**Figure 4.**
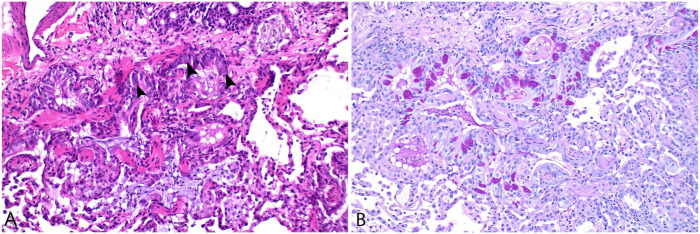
Epithelial damage can result in mucous cell hyperplasia and metaplasia. **A.** Cat lower airways and alveoli; lower airway remodeling with increased mucous cells (arrowheads). H&E. **B.** Cat lower airways and alveoli; mucous cells are apparent with PAS stain.

Variation in mucus composition, amount, and clearance occurs during many respiratory diseases. Epithelial damage can result in hyperplasia of mucous cells in the bronchi and mucosal glands, and in mucous cell metaplasia in the bronchioles. Chronic airway disease can lead to a decreased ability to clear mucus and result in obstruction. Mucous cells can be highlighted with the periodic acid–Schiff (PAS) histochemical stain (**Fig. 4**). This stain typically is not needed to identify mucous cells but can highlight the degree of hyperplasia or metaplasia or identify mucous cells in instances of lower airway remodeling.

## Conducting airways—bronchioles

Bronchi are defined by the presence of cartilage and mucosal glands. These are no longer present after several generations of branching, at which point airways are referred to as *bronchioles*. The *terminal bronchiole* is the most distal bronchiole, with no alveoli arising from the airway. Distal to this, *respiratory bronchioles* have alveoli arising directly from their wall. Some authors define the *transitional bronchiole* as the first respiratory bronchiole between the terminal bronchiole and the more distal respiratory bronchioles.^78^ The number of alveoli within a respiratory bronchiole is described as the degree of “alveolarization.” Respiratory bronchioles with few alveoli are considered to be poorly alveolarized, whereas those with numerous alveoli are well-alveolarized.^70,78^ Respiratory bronchioles have variable numbers of alveoli as a component of the airway wall, and *alveolar ducts* are airways that are completely lined by alveoli. Furthermore, respiratory bronchioles are lined by cuboidal (bronchiolar) epithelium and have smooth muscle in their wall, whereas alveolar ducts are lined by attenuated cells (pneumocytes) and have only scant smooth muscle in their wall. Depending on the species, there may be 1–3 generations of respiratory bronchioles and 2–6 generations of alveolar ducts.^46^ The composition of respiratory bronchioles in many mammals has been reviewed.^72^ Respiratory bronchioles may benefit lung function by adding additional alveoli to a limited volume of lung tissue. To our knowledge, the presence or absence of respiratory bronchioles does not otherwise impact the development of, or susceptibility to, various types of lung diseases among species.

**Figure 5.**
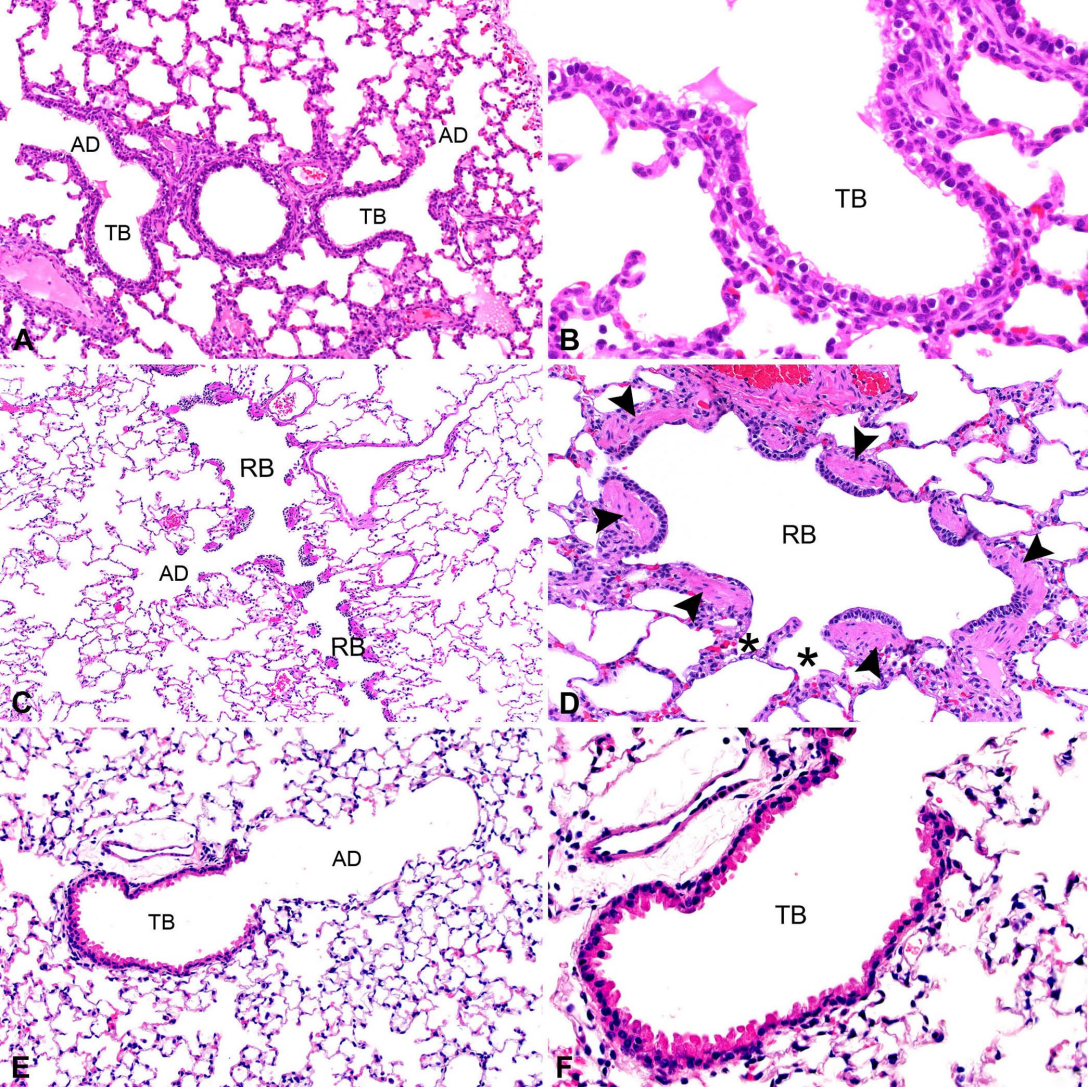
Terminal airways of representative species. **A.** Pig terminal bronchioles (TB) give rise to alveolar ducts (AD) before entering alveoli. **B.** High magnification of a TB, lined by cuboidal epithelium. **C.** Dog respiratory bronchiole (RB) empties into the AD. **D.** Dog, high magnification of RB denoted by the smooth muscle lining (arrowheads) and alveolar outpouchings (*). **E.** Mouse TB empties directly into the AD. **F.** Mouse, high magnification of TB, lined by both club cells and ciliated epithelium.

Cats and dogs have several generations of respiratory bronchioles. Respiratory bronchioles are absent or infrequent and short in horses, cattle, sheep, and pigs, and are poorly developed in mice and rats (Table 1; **Fig. 5**, Suppl. Figs. 1–8).^35,70,72^ Adult rats have diminutive respiratory bronchioles that connect the terminal bronchiole and alveolar ducts. In animals without respiratory bronchioles, terminal bronchioles end directly in alveolar ducts (Fig. 5; Suppl. Figs 1–8).^70^

Bronchioles are composed of *respiratory mucosa* and *adventitia*. The bronchiolar mucosa includes the epithelial layer as well as the lamina propria with its longitudinally arranged elastic fibers and spirally arranged smooth muscle cells.^84^ Distally, airways distribute air to promote efficient gas exchange.^38,78^ Bronchioles are lined by ciliated cells, club cells, mucous cells, neuroendocrine cells, and brush cells. Proximal bronchioles are lined by columnar-to-cuboidal epithelial cells, terminal bronchioles are lined by cuboidal epithelial cells, and respiratory bronchioles are lined by squamous and cuboidal epithelial cells.^53^

Ciliated, mucous, neuroendocrine, and brush cells have been described in the previous section. *Club cells*, formerly called “Clara” cells, are columnar with an invaginated central nucleus and extend more apically than ciliated cells. They are most abundant in bronchioles but have been found more proximally in the rat and mouse.^5^ Club cells are stem cells that aid in repairing epithelium, maintaining homeostasis, metabolizing toxins, and performing secretory functions.^9^ Ultrastructural studies describe highly variable morphologic characteristics and organelle composition of nonciliated cells between species; however, most references describe all nonciliated bronchiolar epithelial cells as club cells.^50,53^ Advanced genomic sequencing technologies could be helpful in further characterizing these cells and determining if they represent unique cell types.^12,61^

In horses, proximal bronchioles contain ciliated and nonciliated cells with rare mucous cells, whereas mucous cells are absent in terminal bronchioles. In sheep, one study described the tracheobronchial epithelium and identified mucosal glands, basal, mucous, and ciliated cells in noncartilaginous airways. Basal and mucous cells were identified in airways that also contained club cells.^30^ This suggests that distinguishing between bronchi and bronchioles can be challenging in some species; sheep may have mucous cells within bronchioles. A separate study described ovine primary and secondary bronchioles that contained basal cells, intermediate cells, ciliated cells, and club cells.^4^ Distal bronchioles of sheep are lined only by ciliated cells and club cells.^4,30^ In mice, basal, ciliated, nonciliated secretory, and brush cells are described in the airway epithelium. Brush cells and basal cells decrease in number in the distal airways.^43^ In rats, nonciliated cells, brush or tuft cells, neuroendocrine cells, and ciliated cells have been identified in small intrapulmonary airways.^20^

Early studies characterized cells within bronchioles of several species using electron microscopy.^49,50,51^ These studies reported on the average numbers of cells across all levels of bronchioles. Generally, ciliated cells become fewer as the airways progress distally within all species.^6^ In cattle, horses, and sheep, on average, without consideration of level of bronchiole, ciliated cells and nonciliated cells are present in approximately equal proportion, with slightly to moderately higher numbers of nonciliated cells.^50,53^ In terminal bronchioles of horses, nonciliated cells are present in a ratio of 4 to 1, according to one study.^71^ In pigs, ciliated and nonciliated cells are present in variable numbers.^3^ In mice and rats, nonciliated cells are >50% of the epithelial cells and ciliated cells are <50% of the epithelial cells within bronchioles.^36,38,53^ In species with a substantial respiratory bronchiolar region (dogs and cats), epithelial populations are predominantly nonciliated in both the nonrespiratory and respiratory bronchioles.^50^ In dogs, proximal bronchioles are lined by numerous ciliated cells, whereas few ciliated cells are found in the terminal bronchioles. Rare ciliated cells were identified in the first generation of canine respiratory bronchioles in one study, and no ciliated cells were found in the second generation of canine respiratory bronchioles and more distally.^50,63^

## Gas-exchange regions

The gas exchange region of the lung is made up of *respiratory bronchioles, alveolar ducts*, and *alveoli.* Alveoli are sac-like, thin-walled structures consisting of a network of capillaries surrounded by interstitial connective tissue and alveolar epithelial cells. Their morphology serves to pack a very large surface area into the small available space within the thoracic cavity. To distinguish them from alveoli arising from the walls of respiratory bronchioles or alveolar ducts, the clusters of alveoli found at the termination of the alveolar duct are sometimes termed *alveolar sacs*, with walls formed entirely of interalveolar septa.

Whereas the alveolar ducts expand during inspiration, expansion of alveoli is considered to be minimal, and gas enters the alveoli mainly by diffusion rather than by convection of bulk air flow.^76,78^ This point is particularly relevant to the deposition of inhaled particles by impaction or gravitational settling, which occurs mainly in the conducting airways rather than in the alveoli. Thus, at the earliest stage of infection from inhalation of aerosolized particles, the bronchioles are primarily affected, until the pathogen replicates and moves to the alveoli.

*Cell types in mammalian alveoli* are type I and type II pneumocytes (alveolar epithelial cells), fibroblasts or myofibroblasts and pericytes (interstitial cells), endothelial cells, and alveolar macrophages, as well as interstitial macrophages, dendritic cells, lymphocytes, and (in some species), pulmonary intravascular macrophages.^6,47^ It is possible to identify subsets or phenotypes for many of these cell types using single-cell transcriptomics and other methods. Studies in domestic animals have suggested 5 endothelial cell types, 6 fibroblast cell types, 6 smooth muscle cell types, and 13 myeloid cell types, including 8–23 phenotypes of macrophages and dendritic cells^27,57^ and 10–35 lymphoid cell types.^58^ Several studies in mice have identified numerous cell subsets as well.^16,33,54^ However, it should be noted that these are defined by patterns of gene expression and some cells are known to transition from one to another of these phenotypes. The percentage of each cell type and their volume and surface areas are given elsewhere.^47^

Species variation exists in terms of branching patterns and surface area of alveoli, but the cellular composition of the alveolar lining is largely similar.^47^

The *inter-alveolar septum* (synonymous with the alveolar septum, or alveolar wall) is 0.27–0.67-µm thick in different species of mammals.^47^ Collagen and elastic fibers are present but scarce, and can be demonstrated by Masson trichrome and Verhoeff–van Gieson (VVG) stains respectively. Ultrastructurally, one side of the alveolar septum is typically thickened by the presence of these scant fibers, whereas the opposite side of the septum is thin, with only a fused basement membrane between the pneumocyte and the endothelial cell. These alternating thick and thin areas along the alveolar septum are thought to balance the need for gas exchange (in the thin areas) and structural support (in the thick areas). However, this difference cannot be appreciated in routine histologic sections. These fibrous and elastic fibers in the alveolar wall connect to form a ring around the opening between the alveolar duct and the alveolus, where they are accompanied by a small ring of smooth muscle. They then continue in the wall of the alveolar duct to reach the fibers that course longitudinally in the airways. In the opposite direction, the fibers in the alveolar septa connect to those in the interlobular septa and pleura. This structural support system allows expansion of the gas exchange regions when the chest wall is pulled outward by the muscles of respiration. This system in mammals combines the ventilator and gas exchanger in a single unit, in contrast to the separate systems in the avian lung.^76,77^

The surface of the inter-alveolar septum is covered by *membranous type I pneumocytes* and *cuboidal type II pneumocytes* (also known as alveolar type 1 and 2 cells, respectively). Type I pneumocytes have such attenuated cytoplasm (~0.33-μm thick in domestic animals^47^) that only their nuclei are routinely discernable in H&E-stained histologic sections. The type I pneumocyte is considered to be the broadest cell in the body, covering an average estimated surface area of 5,098–6,938 μm^2^ in humans, 3,794 μm^2^ in dogs, and 10,157 μm^2^ in horses.^62,77^ A single alveolus is covered by at least 2 different type I pneumocytes, each of which in humans extends through inter-alveolar pores to partially line more than one alveolus. Type II pneumocytes are as frequent as type I pneumocytes but cover only 1/30th of the surface area. Thus, they appear as cuboidal cells tucked into the corners of the alveoli to minimize their negative impact on gas exchange.^77^ They can be distinguished from alveolar macrophages by their close association with the alveolar wall, foamy cytoplasm from intracellular lipid droplets, and anisokaryosis when reactive and proliferating.

When air is transferred among alveoli via pathways that bypass main airways, this is referred to as *collateral ventilation*.^64^ In humans, these alternative pathways can help to reduce the impact of certain disease conditions by helping to maintain oxygen delivery.^64^ Collateral pathways include interalveolar pores (pores of Kohn), interbronchiolar canals (canals of Martin), and bronchiolar-alveolar channels (channels of Lambert).^64^ Interalveolar pores are ∼3–13-μm spaces within alveolar walls, allowing passage between adjacent alveoli. Interbronchiolar canals are ∼30-μm and connect bronchioles; bronchiolar-alveolar channels are ∼120-μm connections between terminal or respiratory bronchioles and alveoli. Interalveolar pores have been identified in horses,^48^ dogs,^32^ cats,^1^ mice,^7^ and rats,^80^ but are rare in pigs and cattle.^17,29^ Interbronchiolar canals have been identified in dogs.^31,32^ In functional measurements of collateral ventilation, cattle and pigs had no collateral ventilation; dogs, horses, and sheep had collateral ventilation.^25,59^ Because resistance to collateral airflow is normally higher than airway resistance, collateral ventilation is thought to be minimal at rest. However, pulmonary acini with airway obstruction develop locally high carbon dioxide and low oxygen concentrations that seem to promote collateral ventilation. This may prevent the development of atelectasis and ensure that different alveoli maintain similar intraluminal pressures. In species without collateral ventilation, ventilation-perfusion balance is thought to be achieved through arterial vasoconstriction.^10^ Cattle and pigs have few inter-alveolar pores (in <10% of the alveolar septa),^29^ which may explain the tendency of these species to develop a lobular pattern of atelectasis or pneumonia, with affected lobules alongside unaffected lobules.^59^

## Circulation

Familiarity with the dual blood supply of the lung—that is the pulmonary and bronchial circulation—is essential to understanding the gross and particularly the microscopic lesions of pulmonary disease. In addition, species differences should be considered when interpreting histomorphology. Recognizing these variations is crucial for accurate histologic interpretation and diagnosis.

## Pulmonary circulation

The pulmonary circulation consists of the pulmonary arteries and pulmonary veins. The pulmonary arteries arise from the bifurcation of the main pulmonary artery. In every species, pulmonary arteries follow along the conducting airways, becoming smaller in caliber to pulmonary arterioles until they enter an acinus. Here they divide to supply a meshwork of pulmonary capillaries within the alveolar walls. The pulmonary veins arise on the other side of the capillary bed. The location of these veins in the lung differs depending on the species.^23^

The pulmonary circulation is a low-resistance, low-pressure system, with high compliance that is well suited to maintaining a balance of pressure and flow to optimize gas exchange. In contrast, the systemic circulation is a high-resistance, high-pressure system designed to generate and sustain the pressure necessary to perfuse tissues throughout the body with oxygenated blood.^8^ Functional differences between the 2 circulatory systems give rise to distinct morphologic features that help veterinary pathologists identify vessels in the pulmonary circulation and the bronchial (systemic) circulation.

**Figure 6.**
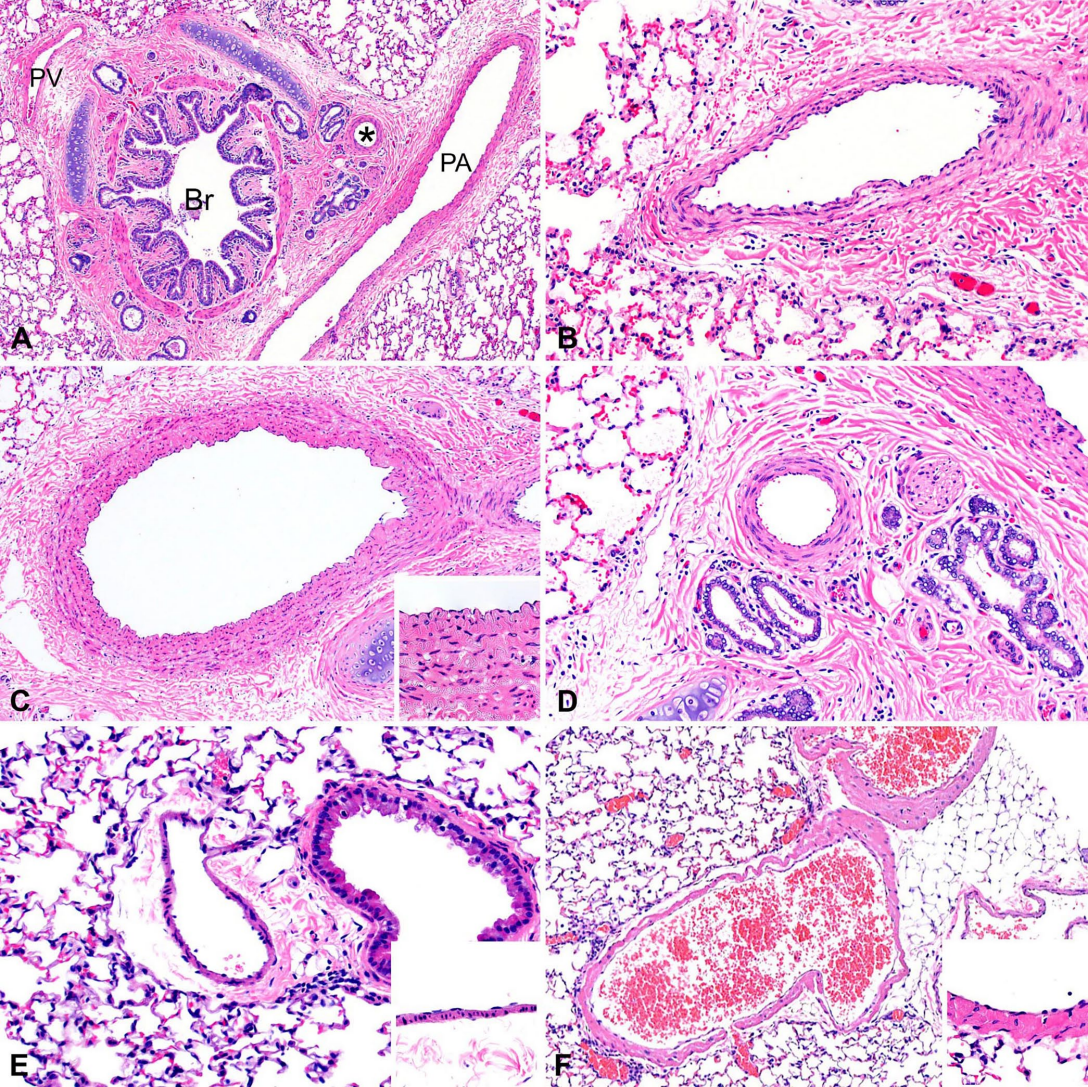
Pulmonary vasculature. H&E. **A.** Sheep pulmonary artery (PA), pulmonary vein (PV), and bronchial artery (asterisk) adjacent to a bronchus (Br). **B.** The pulmonary vein is defined by the thin wall and single layer of elastic lamina. **C.** The pulmonary artery has thick media with noticeable elastic fibers (inset). **D.** Bronchial artery is located next to large airways and mucosal glands. **E.** Mouse pulmonary artery is located adjacent to bronchioles with a thin media (inset). **F.** Mouse pulmonary veins with thick layers of cardiomyocytes (inset).

In contrast to the systemic arteries, pulmonary arteries have a thinner media and a wider lumen with a roughly equal wall-thickness-to-lumen diameter. Pulmonary arteries are classified based on the composition of the media; that is, the presence of elastic lamina and the degree of muscularity.^23,67^ Elastic pulmonary arteries are the capacitance vessels of the lung, and these vessels are identifiable by size, generally having an external diameter >1,000 µm, and by a distinct media. The media has layers of concentric elastic laminae, interspersed with smooth muscle.^23^ Muscular pulmonary arteries and arterioles are the resistance vessels of the lung. These vessels have a defined muscular media delineated by an internal and external elastic lamina. Both elastic and muscular pulmonary arteries have an intima that includes the endothelium and are surrounded by an outer fibrous adventitia (**Fig. 6**).^23^

Species variation exists in the thickness of the muscular media in pulmonary arteries and arterioles. Cattle, pigs, and sometimes cats have very thick muscular pulmonary arteries compared with other veterinary species.^23^ When evaluating feline pulmonary pathology, it is important to be aware of a unique spontaneously arising condition known as pulmonary artery medial hyperplasia, which occurs in a small percentage of cats. This condition was formerly thought to be associated with *Aelurostrongylus abstrusus* and *Toxocara cati* infections. However, because medial hyperplasia occurs in both conventional and SPF cats, the cause of the lesion is unknown. Interestingly, pulmonary hypertension and right ventricular hypertrophy are not associated with this condition.^13,42^ In such feline cases, and similarly in cattle and pigs, it is important to not mistakenly diagnose these thick-walled muscular pulmonary arteries as changes reflective of pulmonary hypertension.

## Histopathology correlate: smooth muscle in pulmonary vasculature

The quantity of smooth muscle present in the media is thought to be a key determinant of the pulmonary vascular response to chronic alveolar hypoxia and may underlie interspecies variability in the development of pulmonary hypertension. Greater medial thickness of small pulmonary arteries is associated with more severe pulmonary hypertension and right ventricular hypertrophy under hypoxic conditions.^69^ This concept is best demonstrated in calves that differ in susceptibility to high-altitude pulmonary hypertension. Calves with thicker muscular pulmonary arteries are more susceptible to developing significant pulmonary vascular remodeling and right-sided heart failure, compared with calves that have thinner pulmonary arteries and that are resistant to disease.^79^ Animals that have adapted to living at high altitudes, such as llamas and yaks, have thinner muscular pulmonary arteries than cattle and are remarkably resistant to high-altitude pulmonary hypertension.^23^

In most mammals, smooth muscle is present in distal pulmonary arterial vessels to an external diameter of ~20 µm, also referred to as arterioles. Of notable exception, goat arterioles are similar to human arterioles in that muscular pulmonary arteries terminate at an external diameter of ~100 µm, continuing as non-muscular pulmonary arterioles. In humans, the finding of significant numbers of muscular arterioles of <70-µm diameter is considered characteristic of hypertensive pulmonary vascular disease. With the exception of the goat, veterinary pathologists are not able to apply this diagnostic criterion for pulmonary hypertension.^23^

**Figure 7.**
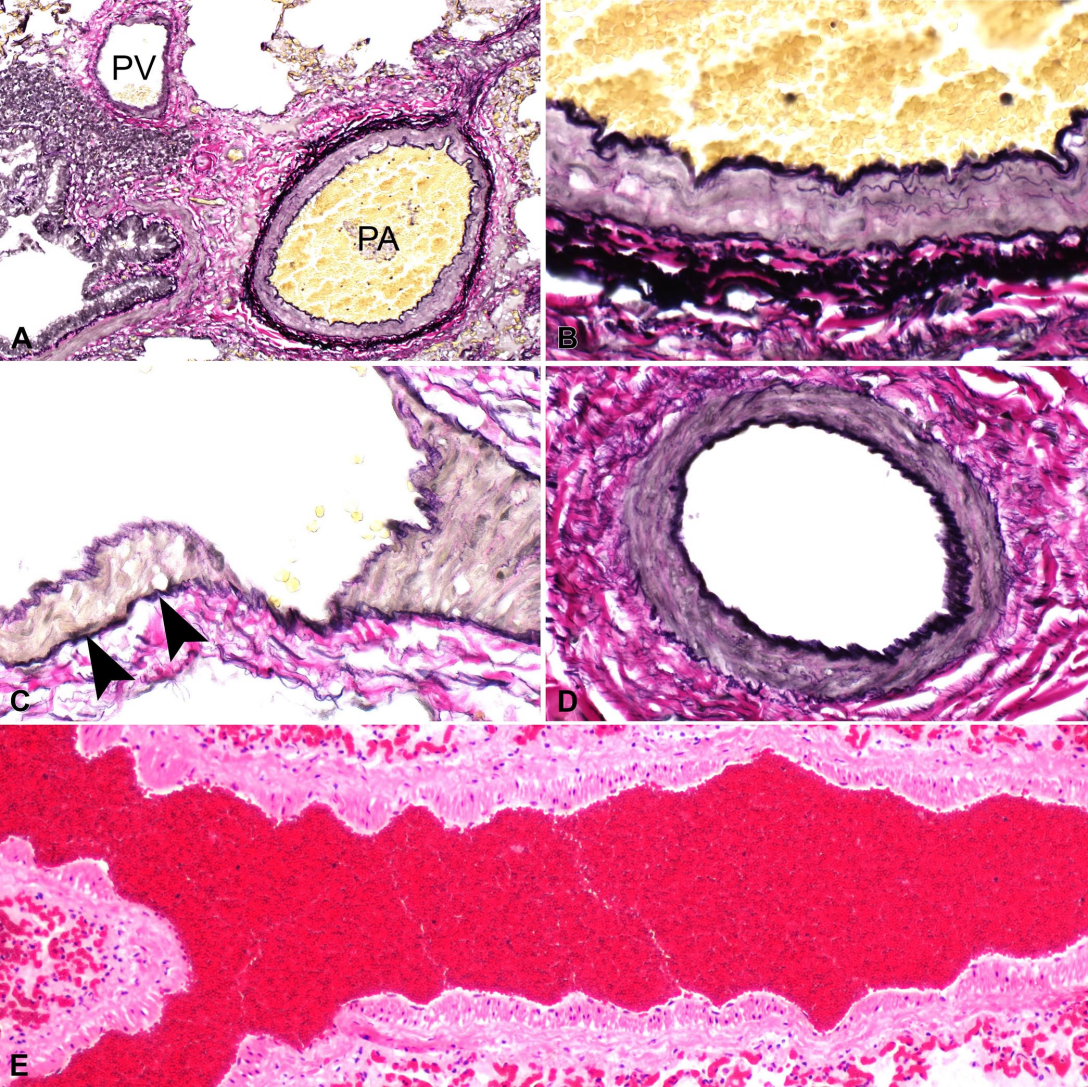
Bovine pulmonary vasculature. **A.** Both pulmonary artery (PA) and vein (PV) are located within the bronchovascular sheath. Verhoeff–Van Gieson (VVG) stain. **B.** Pulmonary artery with internal and external elastic laminae that delineate the tunica media. VVG. **C.** Pulmonary vein with external elastic lamina (arrowheads) but no internal elastic lamina. VVG. **D.** Bronchial artery has an internal elastic lamina but no external elastic lamina. VVG. **E.** Longitudinal section of a bovine pulmonary vein with a beaded appearance due to discrete thick smooth muscle bundles in the intima. H&E.

*Pulmonary veins* have a lower wall-thickness-to-lumen ratio than pulmonary arteries, lack an internal elastic lamina, and have a variably distinct outer elastic lamina that separates the media from the adventitia (Fig. 6). The media consists of an irregular arrangement of elastic fibrils, collagen, and variable amounts of smooth muscle. The relative proportions of these components vary among species. Pulmonary veins are categorized either as thin fibrous, with a poorly demarcated media and adventitia, or muscular, with a distinct elastic lamina between the media and adventitia. The cat, dog, goat, and horse have pulmonary veins with thin fibrous walls; the pulmonary veins in the cow, pig, and rat are more muscular.^23^ Unique to cattle, pulmonary veins have a beaded appearance in their longitudinal section resulting from the thick spiral bundles of smooth muscle in the intima (**Fig. 7**). These bundles of smooth muscle are absent at birth but begin developing at 2 wk of age, becoming fully established by 3 mo.^23,73^ The functional significance of these bundles of smooth muscle is unclear. However, pathologists should take care not to mistake these vessels for pulmonary arteries, as both vessels are located within the bronchovascular bundles. Pulmonary veins of the mouse and rat are unique because the media has a thick layer of cardiomyocytes that is continuous with the left atrium (Fig. 6). In larger vessels, this striated muscle is arranged into an external longitudinal and an internal circular layer.^23^

In addition to their histologic features, pulmonary veins can also be identified by their spatial relationship to surrounding lung structures, a characteristic that varies in a species-specific manner. The pulmonary veins are found in the bronchovascular bundle in the bovine, porcine, ovine, rat, and mouse lung. In contrast, in dogs and cats, pulmonary veins are found independent of the bronchovascular bundle, isolated in the alveolar parenchyma. In the horse, pulmonary veins can be found in the bronchovascular bundle, as intralobular branches within the pulmonary parenchyma, and within interlobular septa and pleura (**Table 3**).^23,67^

**Table 3.** Comparison of the lung vasculature among several species of veterinary importance.

Pleural/septal blood supply	Cattle	Sheep	Pig	Dog	Cat	Mouse	Rat	Horse
	BA	BA	BA	PA	PA	BA	BA	BA
Brachial artery distribution								
Pleura and septa	+	+	+	−	−	−	−	+
Alveoli	−	−	−	−	−	−	−	+
Bronchioles	+	+	+	+	+	−	+	+
Bronchi, hilar lymph node, vasa vasorum of PA & PV	+	+	+	+	+	+	+	+
BA-PA anastomoses (+ near hilum, ++ airways & pleura, +++ alveolar capillaries)	++	++	−	−	−	+++	+	++
Pulmonary artery								
PA hypertension from hypoxia	+++	+	+++	+	ND	ND	++	ND
PA muscular (>6%)	+	+	+	+	+	+	+	+
PA muscular to ≤20 μm	+	+	+	+	+	+	+	+
Pulmonary vein								
PV muscular (vs. thin-walled)	+	ND	+	−	−	+	+	−
PV in interlobular septa	−	−	−	NA	NA	−	−	−
PV follows bronchopulmonary sheath	+	+	+	−	−	+	+	+

BA = bronchial artery; ND = not determined; NA = not applicable; PA = pulmonary artery; PV = pulmonary vein.

Although pulmonary arteries and veins can be distinguished by differences in location and composition of the media, these differences can be subtle or even impossible to determine on H&E alone, especially when pulmonary disease is present. Special stains that highlight the elastic lamina, such as the VVG stain, are helpful in determining differences between pulmonary arteries and veins and can be invaluable when assessing the extent of vascular remodeling (Fig. 7).

## Bronchial circulation

In contrast to pulmonary circulation, which is functionally and structurally adapted for gas exchange, the bronchial circulation arises from the systemic arterial system and serves a nutritive role. The origin of the bronchial arteries varies by species. Bronchial arteries can originate from the aorta, the intercostal arteries, or subclavian arteries. The bronchial circulation is a low-capacity, high-resistance system that receives a relatively small fraction of blood ejected from the left ventricle.^8,23^

In all species except rabbits, bronchial arteries supply the bronchi and bronchioles, as well as the hilar lymph nodes and vasa vasorum of pulmonary arteries and veins. The pleura and well-developed interlobular septa are also perfused by the bronchial circulation in species in which these structures are prominent, including cattle, sheep, pigs, and horses. In horses, bronchial arteries may additionally extend into the alveolar parenchyma, providing arteriolar branches to alveolar capillaries. In contrast, in species with a thin pleura, including dogs and cats, the pulmonary arteries (rather than the bronchial circulation) give nourishment to the pleura.^23,34,35^

Histologically, bronchial arteries are most easily identified adjacent to larger airways, appearing as small-caliber vessels embedded within the adventitia of bronchi and larger bronchioles. Apart from their smaller diameter, bronchial arteries can be distinguished from pulmonary arteries by a proportionally thicker media with narrow lumens, reflecting their origin from the systemic circulation and higher intravascular pressures of the system. Unlike pulmonary arteries, which possess both internal and external elastic laminae, bronchial arteries only have a well-defined internal elastic lamina, which can be visualized with special stains such as the VVG stain (Fig. 7).^8^

Although the bronchial and pulmonary circulations function largely independently, anastomoses between the 2 systems are present in most veterinary species, with the exception of pigs, dogs, and cats.^34^ The distribution, number, and size of these anastomoses vary among species. In mice, anastomoses have been identified at the level of the alveolar capillaries.^55^ In the rat, these anastomoses primarily are found at the hilum; in horses, cattle, and sheep, they are found at various levels along the bronchi, bronchioles, and pleura (Table 3).^23^

## Histopathology correlate: lung lobe torsion

**Figure 8.**
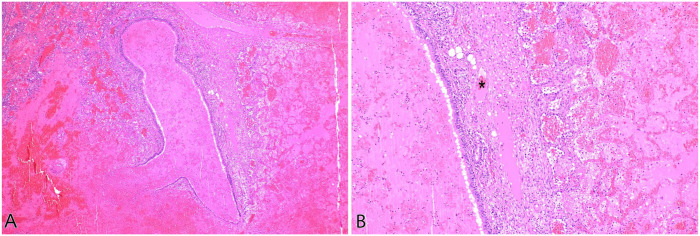
In lung lobe torsion, the low-pressure pulmonary arteries and veins are collapsed, leading to infarction of the alveolar parenchyma; the high-pressure bronchial arteries often remain patent, preserving perfusion to the large airways. Cat, bronchiole and alveoli. H&E. **A.** Viable bronchiolar mucosa surrounded by necrotic and hemorrhagic alveolar parenchyma. **B.** Higher magnification of preserved bronchiolar epithelium, an intact vessel (*), and necrotic alveolar parenchyma.

The systemic origin of the bronchial circulation has important diagnostic implications in cases of lung lobe torsion. In lung lobe torsions, the low-pressure pulmonary arteries and veins are collapsed, leading to infarction of the alveolar parenchyma. However, the high-pressure bronchial arteries often remain patent, preserving perfusion to the large airways. This results in a characteristic histologic finding of viable bronchial mucosa surrounded by necrotic and hemorrhagic parenchyma (**Fig. 8**). Recognition of this pattern can aid in the diagnosis of lung lobe torsion via antemortem lung biopsy or postmortem examination. This condition is more common in dogs but is also reported in cats.^66^ Although unproven and not yet observed, it is reasonable to hypothesize that in species with extensive bronchopulmonary anastomoses, or direct bronchial supply to the alveoli, collateral perfusion could mitigate parenchymal necrosis, depending on the extent and functionality of these connections.

## Lung types in domestic animals, mice, and rats

We have described structures of the lungs with mention of species-specific information. To synthesize this information, we offer the following summary of species comparisons. The following 4 categories define lung morphology based on subgross anatomy (Tables 1, 3).^23,34,35,59^ Each species has minor variations from these patterns as well as unique features not included in the classification.

- *Type I* (found in cattle, pigs, and sheep) has well-developed secondary lobules with thick interlobular septa and pleura. It has few or no respiratory bronchioles. The pulmonary artery and vein are in close proximity within the bronchovascular sheath. A bronchial artery supplies the interlobular septa and pleura and, separately, the airway walls and associated vessels, but it ends at the terminal bronchiole. The pulmonary artery supplies the alveolar ducts and alveoli. Anastomoses exist between the bronchial and pulmonary arteries.
- *Type II* (found in dogs and cats) lacks secondary lobules with inapparent interlobular septa. It has a thin pleura. Respiratory bronchioles and large alveolar ducts are present. The pulmonary vein is separate from the pulmonary artery, which is within the bronchovascular sheath. A bronchial artery supplies the airway walls and the pulmonary artery and vein, but it ends at the respiratory bronchiole. No anastomoses exist between the bronchial and pulmonary arteries.
- *Variant type II* (found in mice and rats) has an absence of secondary lobules with inapparent interlobular septa. It has a thin pleura. Respiratory bronchioles and large alveolar ducts are absent. The pulmonary artery and vein are in close proximity within the bronchovascular sheath. A bronchial artery does not supply the pleura. It does supply the airway walls in mice and rats (but not rabbits). Low numbers of anastomoses exist between the bronchial and pulmonary arteries.
- *Type III* (found in horses) has moderately developed secondary lobules with thick interlobular septa and pleura. Respiratory bronchioles are occasional. The pulmonary artery and vein are in close proximity within the bronchovascular sheath in the distal lung tissue. A bronchial artery supplies the pleura (via both the interlobular septa and directly from hilus to pleura) and, separately, the airway walls and associated vessels. Both the bronchial artery and pulmonary artery continue from the bronchovascular sheath to the bronchioles, alveolar ducts, and alveoli. Rare anastomoses exist between the bronchial and pulmonary arteries.

The microanatomy of various species commonly analyzed in diagnostic and experimental pathology has been extensively characterized using ultrastructural and light microscopy. Understanding normal anatomy aids in recognizing pathologic alterations and leads to the discovery of mechanisms underlying disease.

## Supplemental Material

sj-pdf-1-vdi-10.1177_10406387251413159 – Supplemental material for Microscopic anatomy of the lungs of domestic animals, mice, and rats Supplemental material, sj-pdf-1-vdi-10.1177_10406387251413159 for Microscopic anatomy of the lungs of domestic animals, mice, and rats by Kathleen R. Mulka, Rachael C. Gruenwald, Tzushan Sharon Yang and Jeff L. Caswell in Journal of Veterinary Diagnostic Investigation
