# Supplementary material for: Microscopic anatomy of the lungs of domestic animals, mice, and rats
Source: J Vet Diagn Invest. 2026 Jan 21:10406387251413159. Online ahead of print. doi: 10.1177/10406387251413159 (PMC12823369; doi:10.1177/10406387251413159)
Supplement: sj-pdf-1-vdi-10.1177_10406387251413159 – Supplemental material for Microscopic anatomy of the lungs of domestic animals, mice, and rats [file sj-pdf-1-vdi-10.1177_10406387251413159.pdf]

Mulka KR, et al. Microscopic anatomy of the lungs of domestic animals, mice, and rats

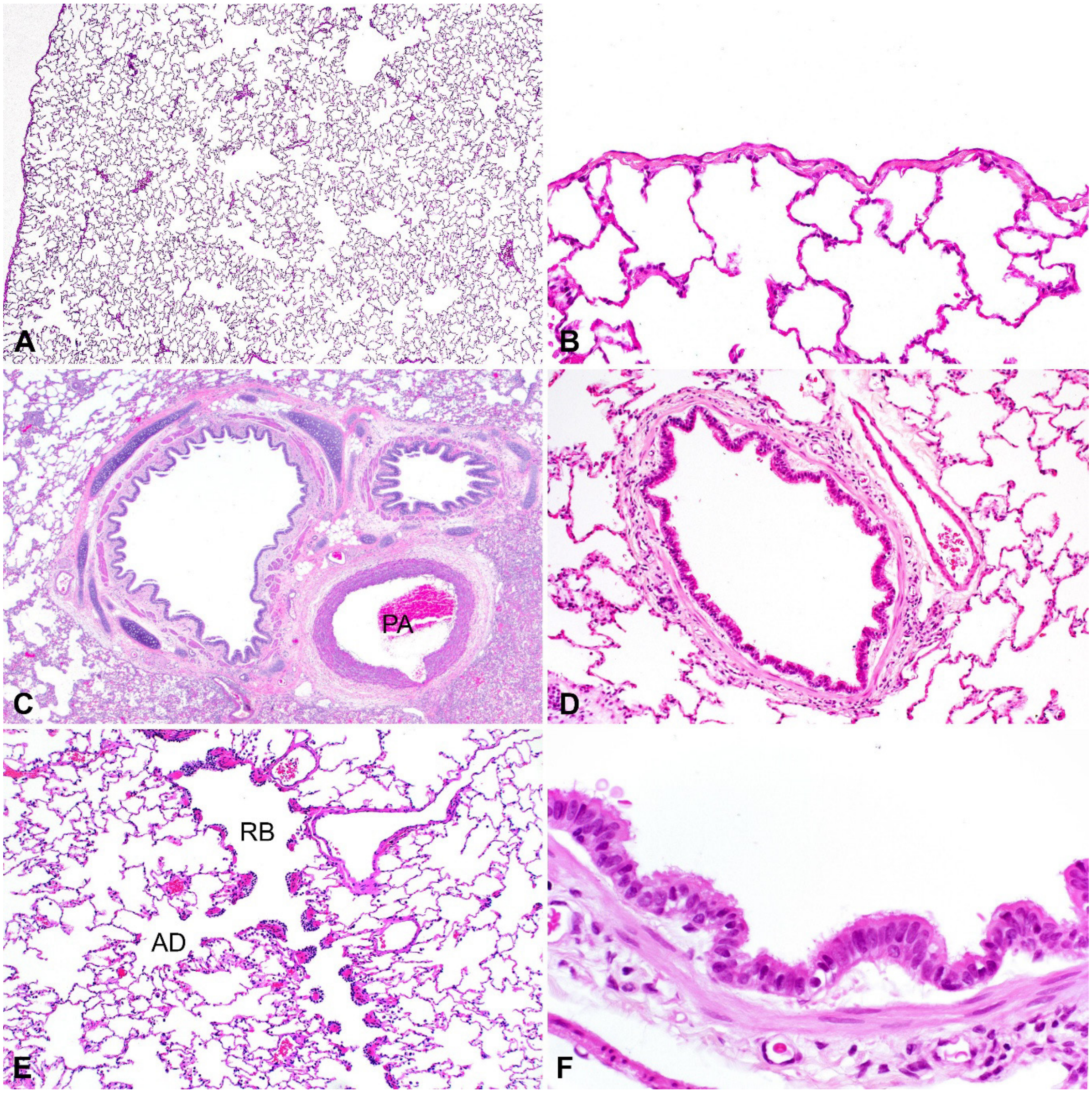

Supplemental Figure 1. Pulmonary structures of the dog. **A.** Pulmonary parenchyma with no interlobular septa. **B.** Pleura is thin. **C.** Bronchus with adjacent pulmonary artery (PA) within the bronchovascular sheath. **D.** Bronchiole with adjacent pulmonary artery. **E.** Respiratory bronchiole (RB) emptying into an alveolar duct (AD). **F.** Bronchiolar epithelium.

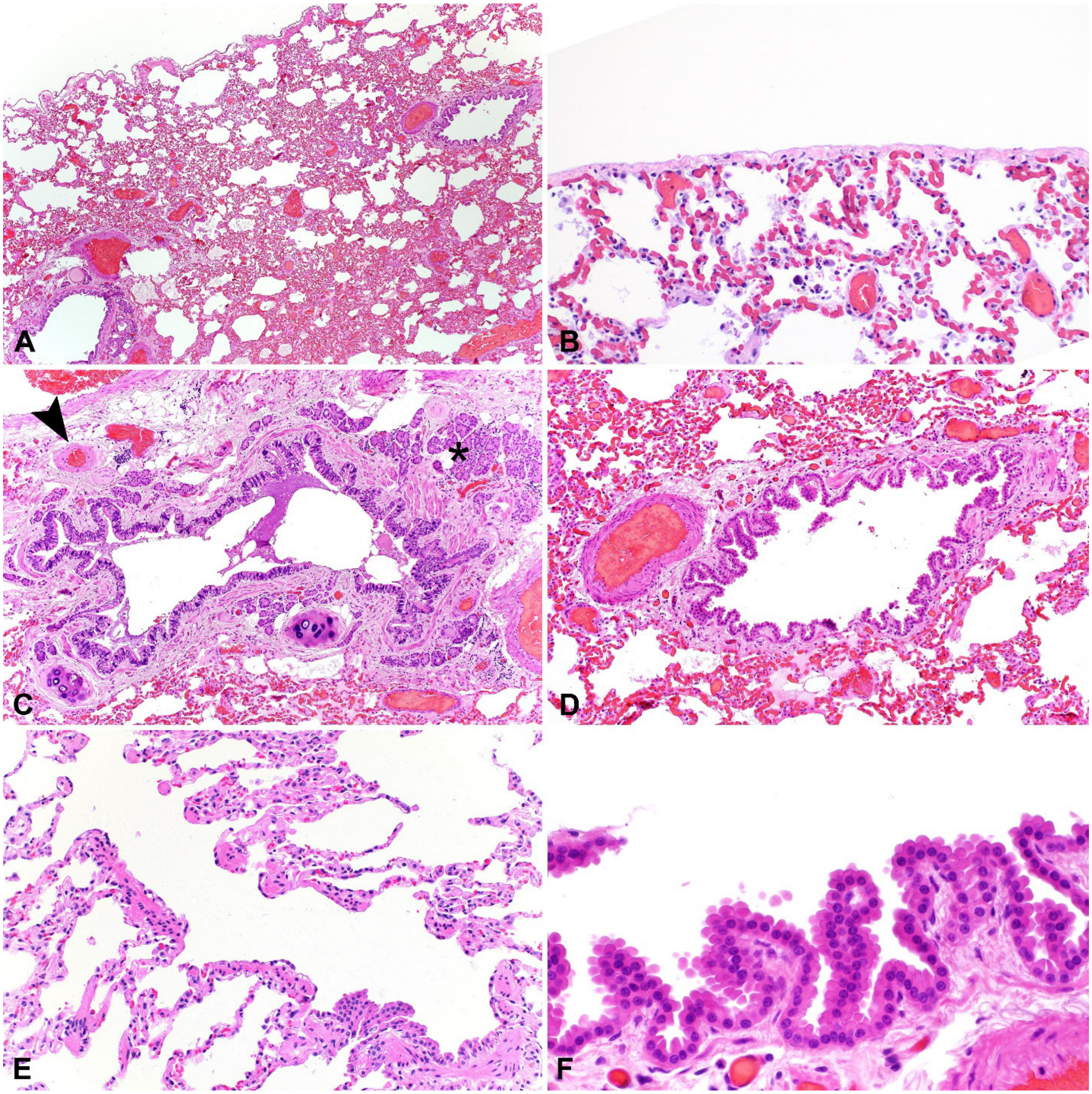

Supplemental Figure 2. Pulmonary structures of the cat. **A.** Pulmonary parenchyma with no interlobular septa. **B.** Pleura is thin. **C.** Bronchus with adjacent bronchial artery (arrowhead) and prominent mucosal glands (asterisk). **D.** Bronchiole with adjacent pulmonary artery. **E.** Respiratory bronchiole with a few alveoli arising from the wall. **F.** Bronchiolar epithelium.

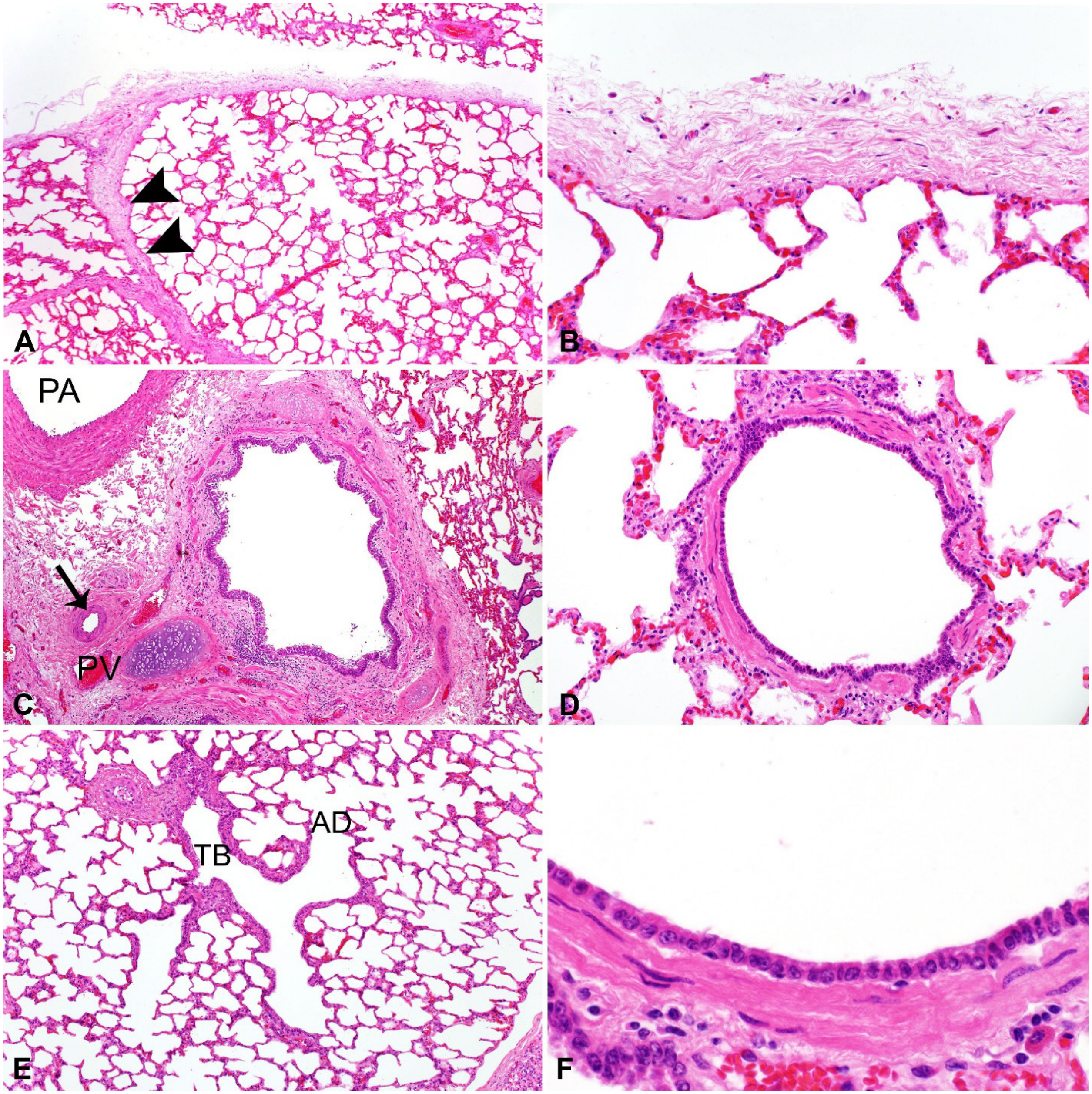

Supplemental Figure 3. Pulmonary structures of cattle. **A.** Pulmonary parenchyma with interlobular septum (arrowheads). **B.** Pleura is thick. **C.** Bronchus with adjacent pulmonary artery (PA), pulmonary vein (PV), and bronchial artery (arrow). **D.** Bronchiole. **E.** Terminal bronchiole (TB) empties into the alveolar duct (AD) before entering the alveoli; no respiratory bronchiole. **F.** Bronchiolar epithelium.

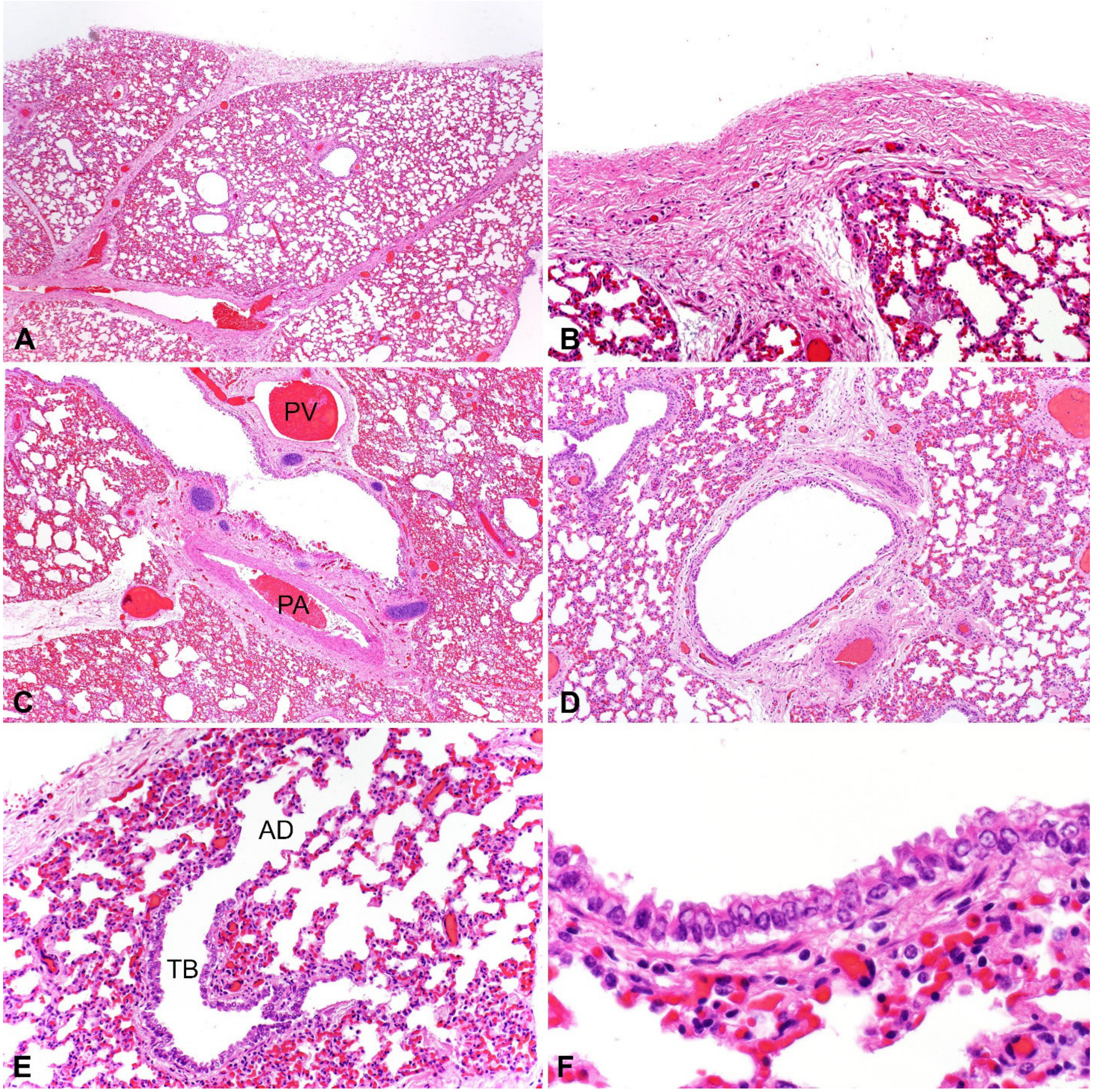

Supplemental Figure 4. Pulmonary structures of the horse. **A.** Pulmonary parenchyma with interlobular septa. **B.** Pleura is thick. **C.** Bronchus with adjacent pulmonary artery (PA) and pulmonary vein (PV). **D.** Bronchiole with adjacent PA. **E.** Terminal bronchiole (TB) empties into the alveolar duct (AD) before entering the alveoli; no respiratory bronchiole. **F.** Bronchiolar epithelium.

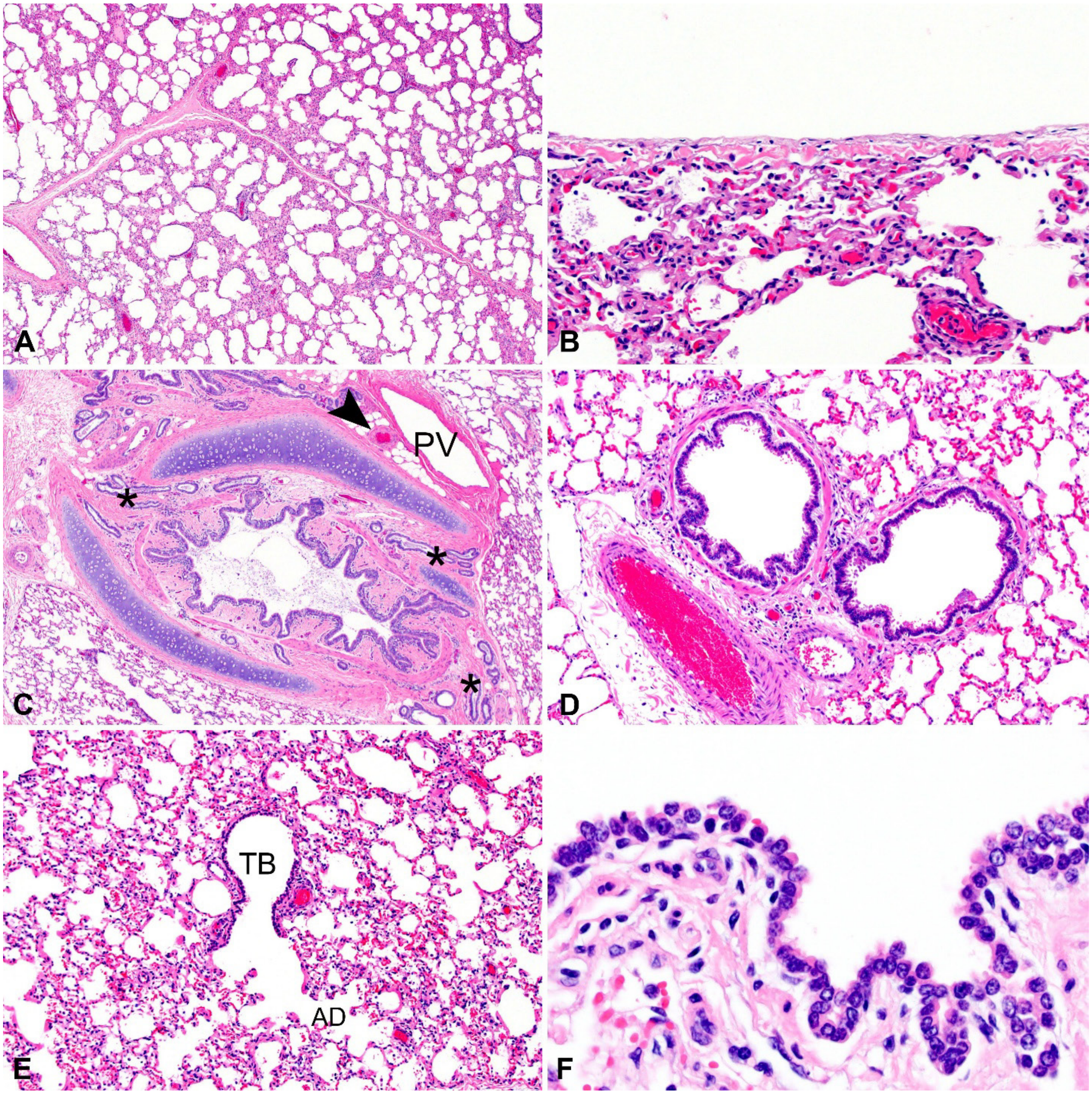

Supplemental Figure 5. Pulmonary structures of sheep. **A.** Pulmonary parenchyma with interlobular septa. **B.** Pleura is of intermediate thickness. **C.** Bronchus with adjacent pulmonary vein (PV), bronchial artery (arrowhead), and mucosal glands (asterisks). **D.** Bronchioles with adjacent pulmonary artery. **E.** Terminal bronchiole (TB) empties into the alveolar duct (AD) before entering the alveoli; no respiratory bronchiole. **F.** Bronchiolar epithelium.

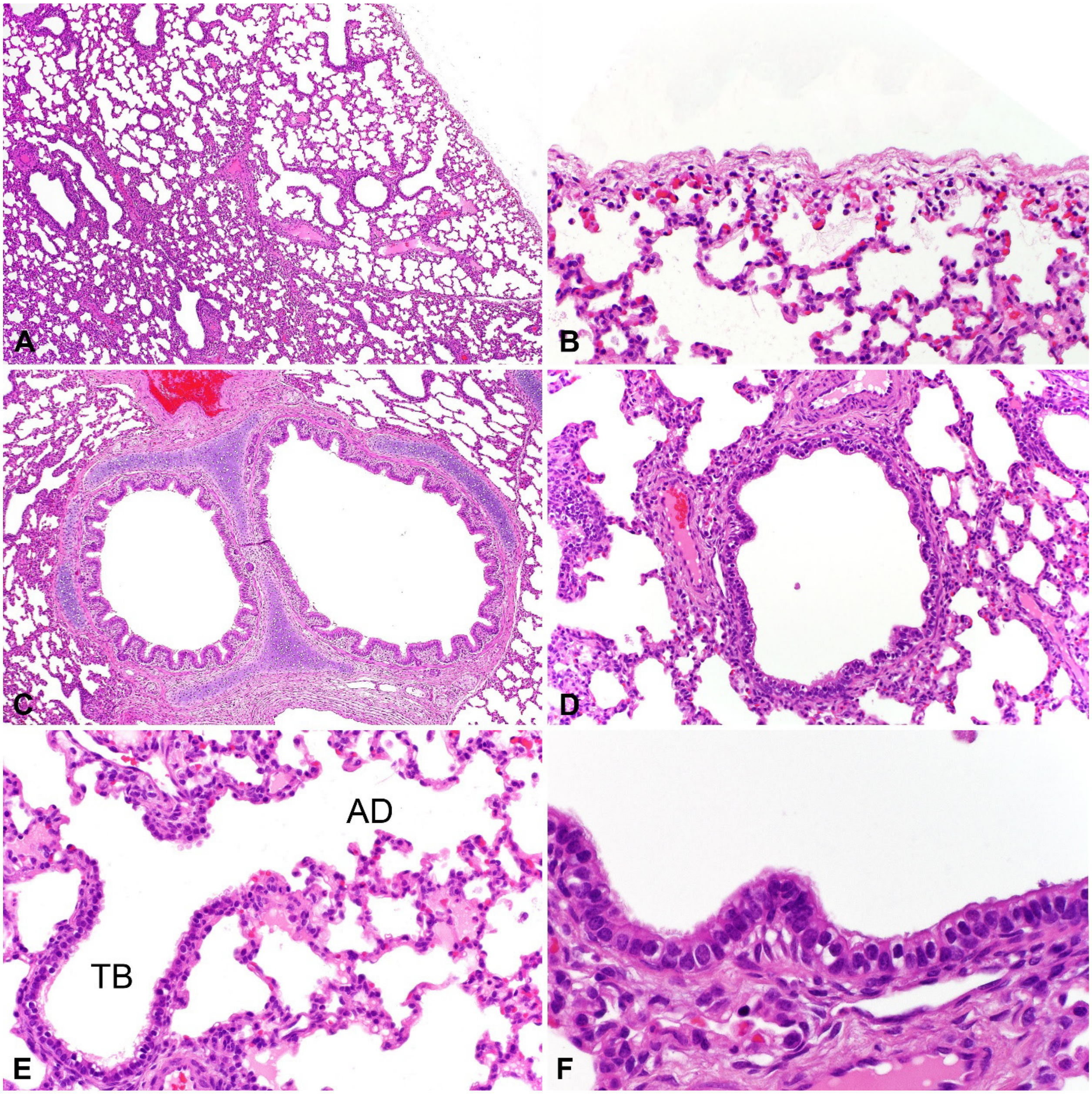

Supplemental Figure 6. Pulmonary structures of the pig. **A.** Pulmonary parenchyma with interlobular septa. **B.** Pleura. **C.** Bronchi with adjacent pulmonary artery. **D.** Bronchiole with adjacent pulmonary artery. **E.** Terminal bronchiole (TB) empties into the alveolar duct (AD) before entering the alveoli; no respiratory bronchiole. **F.** Bronchiolar epithelium.

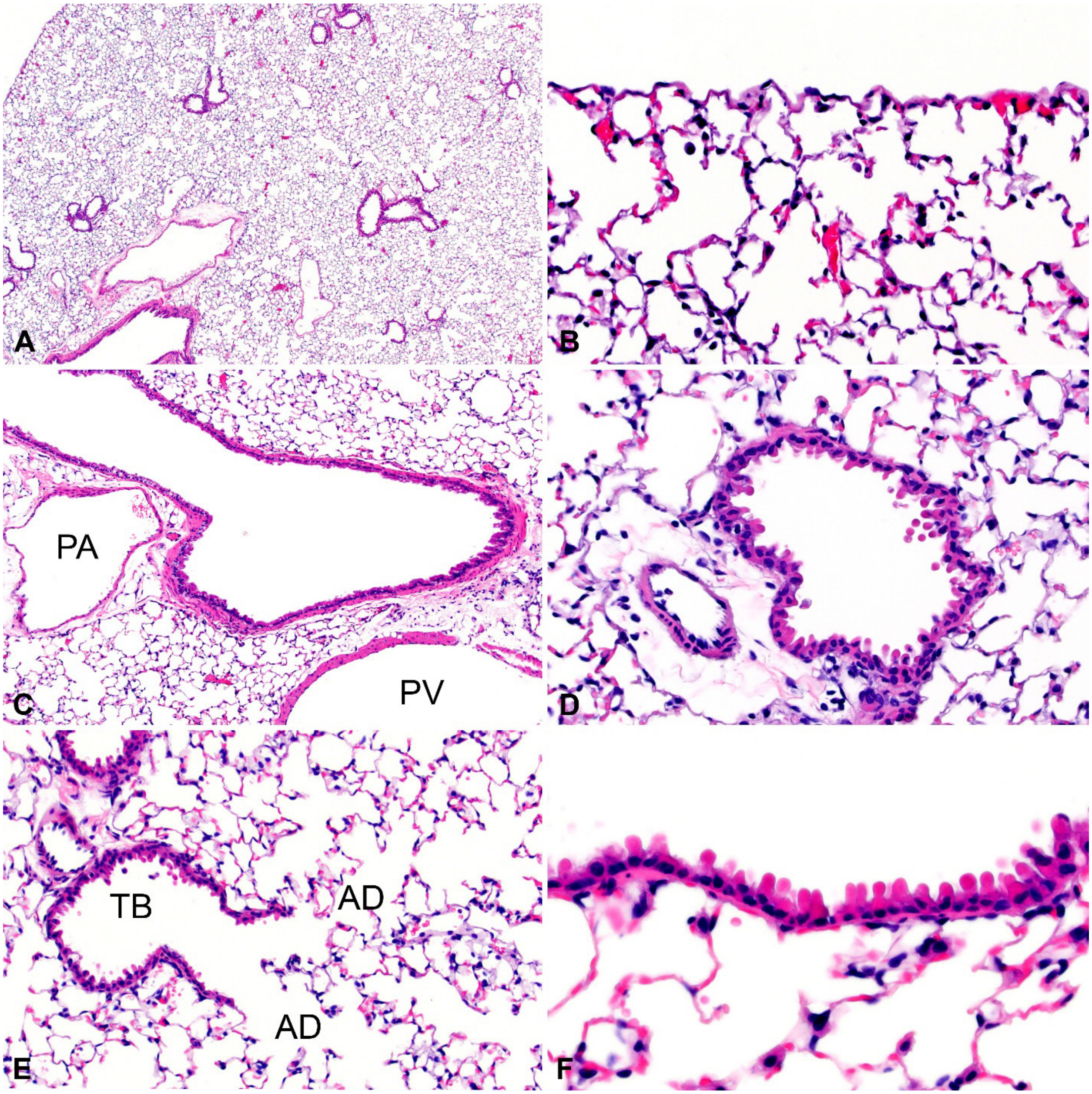

Supplemental Figure 7. Pulmonary structures of the mouse. **A.** Pulmonary parenchyma with no interlobular septa. **B.** Pleura is thin. **C.** Large bronchiole with adjacent pulmonary artery (PA) and vein (PV). **D.** Small bronchiole with adjacent pulmonary artery. **E.** Terminal bronchiole (TB) empties into alveolar ducts (AD) before entering the alveoli; no respiratory bronchiole. **F.** Bronchiolar epithelium.

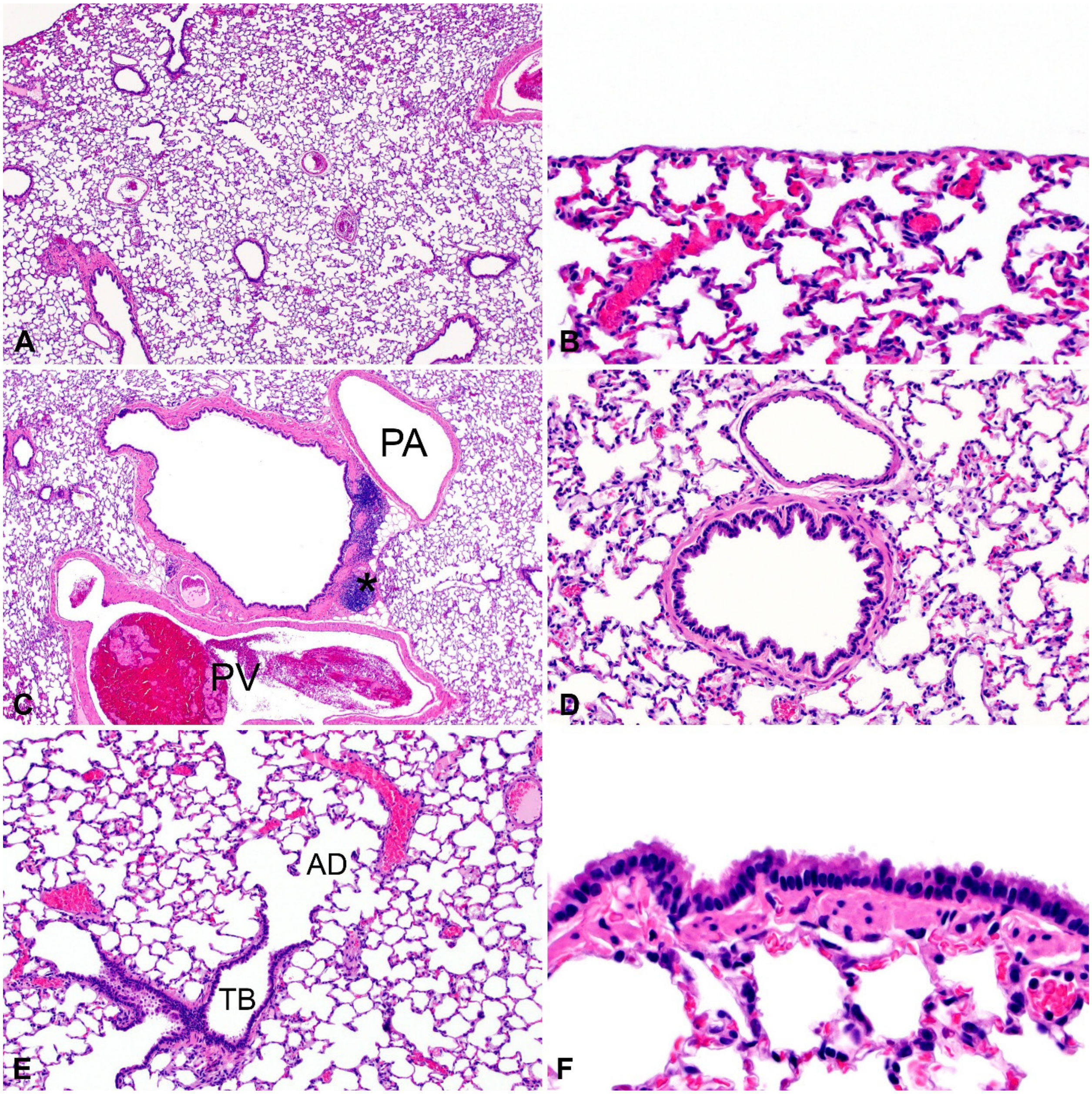

Supplemental Figure 8. Pulmonary structures of the rat. **A.** Pulmonary parenchyma with no interlobular septa. **B.** Pleura is thin. **C.** Large bronchiole with adjacent bronchiole-associated lymphoid tissue (asterisk), pulmonary artery (PA), and pulmonary vein (PV). **D.** Small bronchiole with adjacent pulmonary artery. **E.** Terminal bronchiole (TB) empties into an alveolar duct (AD) before entering the alveoli; no respiratory bronchiole. **F.** Bronchiolar epithelium.
